# Allogeneic Hematopoietic Stem Cell Transplantation for Children With Acute Lymphoblastic Leukemia: Shifting Indications in the Era of Immunotherapy

**DOI:** 10.3389/fped.2021.782785

**Published:** 2021-12-23

**Authors:** Tony H. Truong, Cristian Jinca, Georg Mann, Smaranda Arghirescu, Jochen Buechner, Pietro Merli, James A. Whitlock

**Affiliations:** ^1^Division of Pediatric Oncology, Blood and Marrow Transplant/Cellular Therapy, Alberta Children's Hospital, University of Calgary, Calgary, AB, Canada; ^2^Department of Pediatrics, Victor Babeş University of Medicine and Pharmacy, Timişoara, Romania; ^3^Children's Cancer Research Institute, St. Anna Children's Hospital, Vienna, Austria; ^4^Department of Pediatric Hematology and Oncology, Oslo University Hospital, Oslo, Norway; ^5^Department of Pediatric Hematology/Oncology, Cell and Gene Therapy, Bambino Gesù Children's Hospital, IRCCS, Rome, Italy; ^6^Department of Paediatrics, Hospital for Sick Children/University of Toronto, Toronto, ON, Canada

**Keywords:** hematopoietic stem cell transplant (HSCT), acute lympoblastic leukemia, indications and outcome, immunotherapy, children, pediatrics, B-ALL, relapse

## Abstract

Pediatric acute lymphoblastic leukemia generally carries a good prognosis, and most children will be cured and become long-term survivors. However, a portion of children will harbor high-risk features at the time of diagnosis, have a poor response to upfront therapy, or suffer relapse necessitating more intensive therapy, which may include allogeneic hematopoietic stem cell transplant (HSCT). Recent advances in risk stratification, improved detection and incorporation of minimal residual disease (MRD), and intensification of upfront treatment have changed the indications for HSCT over time. For children in first complete remission, HSCT is generally reserved for those with the highest risk of relapse. These include patients with unfavorable features/cytogenetics who also have a poor response to induction and consolidation chemotherapy, usually reflected by residual blasts after prednisone or by detectable MRD at pre-defined time points. In the relapsed setting, children with first relapse of B-cell ALL are further stratified for HSCT depending on the time and site of relapse, while all patients with T-cell ALL are generally consolidated with HSCT. Alternatives to HSCT have also emerged over the last decade including immunotherapy and chimeric antigen receptor (CAR) T-cell therapy. These novel agents may spare toxicity while attempting to achieve MRD-negative remission in the most refractory cases and serve as a bridge to HSCT. In some situations, these emerging therapies can indeed be curative for some children with relapsed or resistant disease, thus, obviating the need for HSCT. In this review, we seek to summarize the role of HSCT in the current era of immunotherapy.

## Introduction

Childhood acute lymphoblastic leukemia (ALL) is one of the most curable cancers in pediatric oncology, with 80–90% of children surviving into adulthood (1, 2). It was recognized early on that features at the time of ALL presentation, namely, age and leukemia burden (white blood count or peripheral blood blast count), confer varying degrees of treatment success, such that patients could be stratified into different groups (3, 4). As understanding of prognostic factors increased, other ALL features such as central nervous system (CNS) involvement, immunophenotype, cytogenetics, early response to therapy, and end of induction response, including the presence of measurable/minimal residual disease (MRD), became incorporated into such risk groupings (5, 6). This formed the foundation for risk stratification in ALL diagnosis. Correspondingly, treatment intensity could be modified based on risk status, therefore, increasing the chance of cure in the highest risk patients while minimizing long-term toxicity in those with the lowest risk.

Hematopoietic stem cell transplantation (HSCT) is a highly effective treatment for ALL, but given both acute and long-term complications, it is usually reserved for patients with high-risk features in first complete remission (CR1), refractory or relapsed disease. As upfront treatment for newly diagnosed ALL has improved and evolved over the last few decades, so too have indications for the use of HSCT. Similarly, as techniques to detect response to treatment have become more sensitive over time with the incorporation of MRD, HSCT indications have also been updated accordingly. Finally, with the emergence of highly effective immunotherapy and immune effector cellular therapies such as chimeric antigen receptor (CAR) T-cells, the timing, and even the role, of HSCT is changing.

Both the American Society for Transplantation and Cellular Therapy and the European Society for Blood and Marrow Transplantation have produced expert-guided consensus documents outlining recommendations for HSCT in pediatric ALL (7, 8); however, practices may be influenced by patient status, donor availability, and emerging data from recent clinical trials. Overall, allogeneic HSCT has been considered as the standard of care for pediatric patients with high-risk ALL in first complete remission (CR1) and in second CR (CR2). The use of HSCT in patients beyond CR2 is less clear, due to the increased risk and decreased efficacy in this setting, and with the emergence of alternative potentially curative therapies such as CAR T-cell therapy or other investigative agents. Tisagenlecleucel, a CAR T-cell therapy, has been approved as the standard of care for CD19-positive pediatric ALL patients with primary refractory/resistant disease who failed two lines of therapy or those with second or greater relapse (US, Canada, and Europe) and any relapse after HSCT (Canada and Europe only) (8–10). However, the need for subsequent HSCT as consolidation therapy is dependent on factors such as the presence of specific co-stimulatory domains and the persistence of CAR T-cells post-infusion. Please see the companion paper on CAR T-cells by Buechner et al. in this Frontiers series.

The improvement in ALL outcome over time is directly related to multi-center collaboration within large cooperative groups and the development of consecutive trials that build upon prior knowledge. Despite variability in patient populations (e.g., inclusion criteria), definitions of risk-group stratification, treatment delivered, and assessment of response, common principles have emerged to better define high-risk patients with ALL. In this paper, we summarize the collective experience of large cooperative groups from North America and Europe, which have advanced the treatment of newly diagnosed and relapsed ALL. We recognize the valuable contribution of other study groups, including the Japanese Pediatric Leukemia Study Group, and other countries that have participated in collaborative studies around the world. In advancing care, various HSCT indications have been developed among cooperative groups, which, although varied, have common elements which will be highlighted.

## Newly Diagnosed B-Cell Acute Lymphoblastic Leukemia

### North American Study Groups

Among patients with newly diagnosed B-cell precursor (BCP)-ALL, high-risk features that portend a poor prognosis include unfavorable cytogenetics and positive MRD at the end of induction (EOI) (6, 11). Historically, poor cytogenetics including hypodiploidy, defined as <44 chromosomes, and t(9;22)/Philadelphia chromosome (Ph+)-ALL were indications for HSCT in CR1. However, with the advent of MRD and the intensification of chemotherapy for those with MRD positivity, patients with hypodiploidy are no longer routinely treated with HSCT in CR1. In successive St. Jude Total Therapy studies, patients with hypodiploid ALL who achieved negative MRD at EOI had a 5-year event-free survival (EFS) of 85 vs. 41% for those who did not, indicating that chemotherapy alone was sufficient to cure patients with hypodiploid ALL (12).

Similarly, with Ph+ ALL, the introduction of tyrosine kinase inhibitors combined with intensive chemotherapy has yielded improved outcomes such that these patients are no longer *per se* transplanted in CR1. Long-term survival for these patients treated with intensive chemotherapy approach 80%, with the use of either imatinib or dasatinib (13, 14). Patients with persistent MRD positive disease, however, remain eligible for HSCT. Therefore, early response to therapy as defined by MRD remains the most prognostic factor for high-risk newly diagnosed pediatric Ph+ ALL patients in deciding when to proceed to HSCT. This is discussed further by Vettenranta et al. in this Frontiers series.

Patients who are MRD positive at EOI are at high risk for relapse and proceed to an intensified consolidation. However, among the National Cancer Institute (NCI) standard-risk (SR) patients, the prognostic significance of end of consolidation (EOC) MRD of 0.1% to <1% is currently under active study within the Children's Oncology Group (COG) with the introduction of immunotherapy strategies aimed to avoid HSCT. In a current COG frontline protocol, such patients are treated with an augmented BFM-based regimen with the additional non-random assignment of two cycles of blinatumomab (NCT03914625).

Among NCI high-risk patients, the prognostic value of EOC MRD is more pronounced. AALL0232 showed that when patients with EOI MRD >1% were treated with more intensive therapy, outcomes were highly dependent on EOC MRD. Patients with EOC MRD <0.01% vs. those with ≥0.01% had better 5-year survival of 79 vs. 39%, respectively (15). Currently, these patients meet the criteria to proceed to HSCT; however, some of these patients may be eligible to receive CAR T-cells targeting CD19 (tisagenlecleucel) on a currently open clinical trial (CASSIOPEIA study, NCT03876769) available in North America and Europe.

### Hematopoietic Stem Cell Transplant for B-Cell Precursor-Acute Lymphoblastic Leukemia With Primary Induction Failure

**Figure 1:**
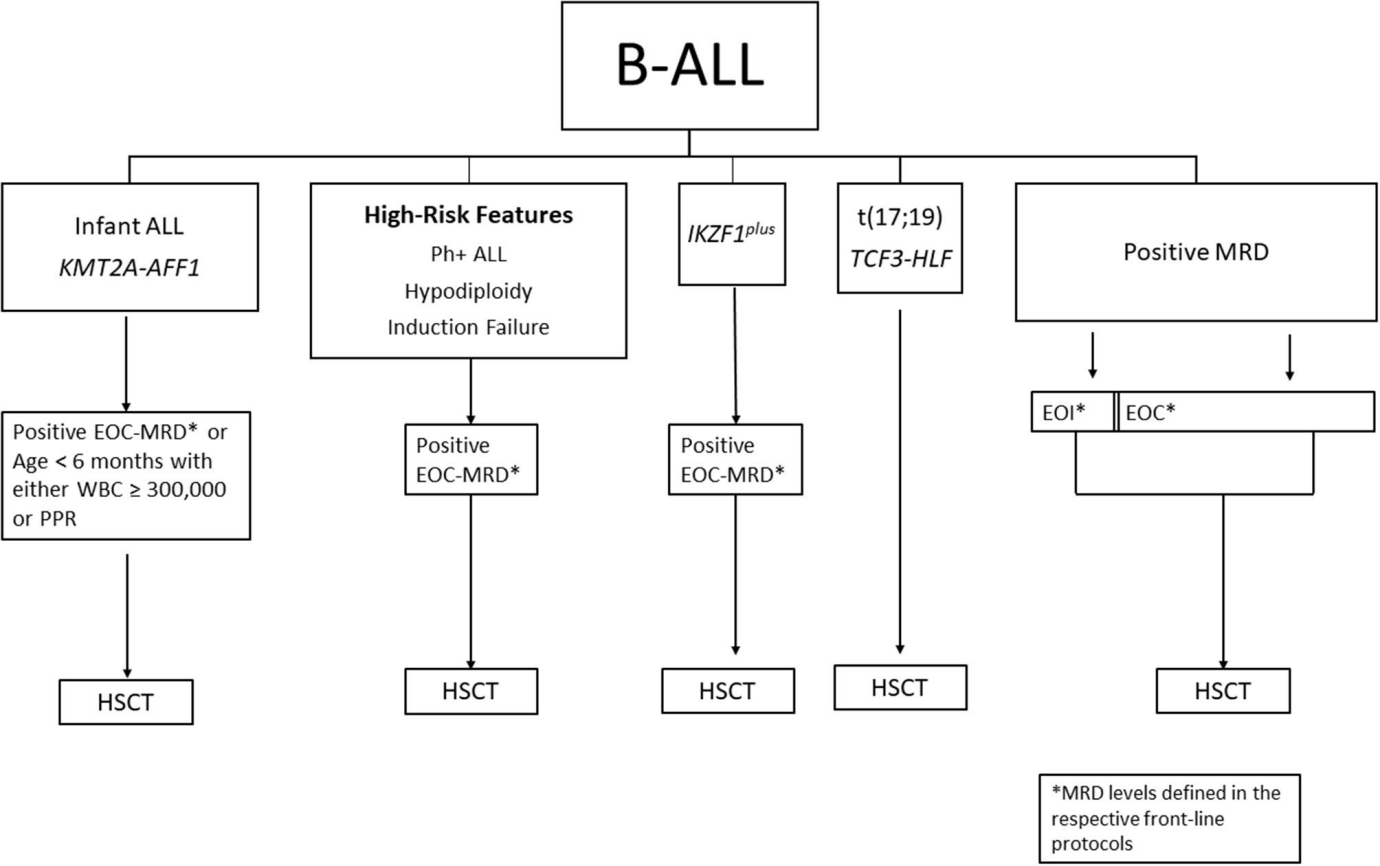
Summary of HSCT considerations for B-ALL in CR1. MRD, minimal residual disease; EOI, end of induction; EOC, end of consolidation; PPR, prednisone poor response; NCI, national cancer institute; HR, high-risk; SR, standard-risk; HSCT, hematopoietic stem cell transplant.

Primary induction failure (IF) is typically defined as the persistence of leukemia blasts (M2 marrow: 5–25% blasts or M3 marrow: >25% blasts) in the bone marrow or extramedullary disease at EOI. Patients with induction failure receive intensified therapy (which may include immunotherapy or CAR T-cells) in an attempt to reduce leukemia burden to achieve remission or MRD negative status. A pooled retrospective analysis from 14 cooperative groups studied over 1,000 patients with IF treated from 1985 to 2000 (16). Among patients with BCP-ALL aged 6 years and older, only matched related donor (MRD)-HSCT was better than chemotherapy (10-year OS 59 vs. 35%, respectively), which was not evident in those under age 6 for whom chemotherapy was superior to HSCT (10-year OS 72 vs. 59%, respectively). With the inclusion of MRD, a small subset of patients with morphologic IF who achieved EOI MRD <0.01% had 5-year EFS of 100% indicating that HSCT could be avoided in certain groups with IF (17). Among the high-risk patients (*n* = 17, M2 and M3 marrows) who underwent HSCT in CR1, outcomes were no different when treated with HSCT vs. without (5-year EFS 41 vs. 56%, respectively). What historically was a common indication for transplant, patients with IF now have other options, such as highly effective immunotherapies with blinatumomab, inotuzomab, or CAR T-cell therapy. A summary of HSCT considerations for B-ALL is shown in Figure 1.

### Berlin–Frankfurt–Munster and Associazione Italiana Ematologia Oncologia Pediatrica Study Groups

Within the development of BFM-AIEOP protocols, high-risk (HR) and very high-risk (VHR) leukemia genetics were first defined by t(9;22)/*BCR-ABL* fusion (Ph+ ALL), and KMT2A-AFF1 [t(4;11), MLL-AF4], followed later on by a low hypodiploid chromosome number, the gene fusion TCF3-HLF [t(17;19)], and last the combination of IKZF1 deletion with any of CDKN2A, CDKN2B, PAX5, and/or PAR1 (CRLF2) in the absence of ERG deletions (IKZF1^plus^). The leukemic cell load was incorporated by the so-called BFM risk factor (BFM RF), taking into account the peripheral blood blast count, liver and spleen size (RF = 0.2 × log (number of peripheral blood blasts/L + 1) + 0.06 × liver + 0.04 × spleen size in cm below the costal margin each) (4). Response kinetic features defined induction failure (IF) as ≥5% leukemic blast cells in the bone marrow after a four-drug induction. A poor prednisone response (PPR) was defined as ≥1,000 blast cells/μL in the peripheral blood (PB) on day 8 of prednisone monotherapy plus one intrathecal MTX administration. Flow cytometry (FCM) detection of ≥10% blasts in bone marrow (BM) at day 15 of induction treatment defined a HR cohort (FCM-MRD d15 HR), excluding ETV6-RUNX1, E2A-PBX, and KMT2A fusions. The leukemia-specific immunogenetic rearrangement detected by polymerase chain reaction (PCR) defined a minimal residual disease (MRD) with a leukemic cell load of ≥5 × 10^−4^ at EOC [time point 2 (TP2)] as HR (PCR-MRD HR). A course with PCR-MRD of ≥10^−3^ at the end of induction therapy [EOI MRD, time point 1 (TP1)], and any MRD positivity at EOC, called slow early response (SER), qualified for HR treatment.

The development of risk stratification and subsequent therapeutic measures in consecutive ALL-BFM protocols of the German–Austrian–Swiss ALL-BFM Study Group included HSCT in CR1 first in the study ALL-BFM 90 (18). The HR criteria were (1) Ph+ ALL, (2) the BFM RF, (3) PPR, and (4) IF.

Based on the results of the study ALL-BFM 86 (4), a VHR subset of patients was defined and qualified for a HSCT from a matched sibling donor only (MSD) by the presence of any of the following features: (1) Ph+ ALL, (2) PPR plus one of the following criteria: T-ALL, co-expression of a myeloid marker, BFM-RF of 1.7 or higher, and/or (3) IF.

In the subsequent protocol ALL-BFM 95, criteria for the allocation to HR were: (1) Ph+ ALL or the translocation KMT2A-AFF1, the latter with a 6-year event-free survival (EFS) in study ALL-BFM 90 of 35%, (18) (2) PPR, and (3) IF. The VHR subset with an indication for HSCT was defined by: (1) Ph+ ALL or KMT2A-AFF1 fusion, (2) PPR plus T-ALL and/or a WBC count of 100,000/μl or greater, and (3) IF. The superiority of HSCT for VHR ALL in CR1 compared with chemotherapy alone (CT) could be shown with 56.7 vs. 40.6% disease-free survival (19). For the subset of VHR T-ALL treated in studies BFM-90 and 95, overall survival (OS) rates at 5 years of 67% with HSCT were superior compared with 47% with CT (20).

For the first time, protocol AIEOP-BFM ALL 2000 incorporated MRD over treatment time into risk stratification. HR disease included (1) Ph+ ALL, (2) KMT2A-AFF1 fusion, (3) PPR, (4) IF, or (5) PCR-MRD HR (21). In addition to the BFM-95, VHR criteria now included PCR-MRD HR which qualified for HSCT. In 2004, during study ALL-BFM 2000, the indication criteria for HSCT in CR1 were adapted to the ALL-SCT-BFM-2003 trial, thus excluding patients with an MRD load of <10^−4^ at EOC except for KMT2A-AFF1 positive leukemias (22). Patients with Ph + ALL and a prednisone good response (PGR) and CR at the EOI were also excluded from an HSCT indication.

Since 2004, patients with Ph+ ALL were treated according to two consecutive protocols, the European study for pediatric Ph+ ALL “EsPhALL,” with the later trial incorporating earlier and longer exposure to imatinib (23, 24). The indication for HSCT in EsPhALL2010 was EOC MRD ≥5 × 10^−4^ (high positive) or EOC MRD <5 × 10^−4^ (low positive) with any detectable MRD level at the end of high-risk block 3. Given the benefit of adding imatinib, transplant practices waned over the course of the EsPhALL2010 leading to the conclusion that earlier and prolonged use of imatinib allowed similar survival while avoiding the use of HSCT.

In the trial AIEOP-BFM 2009, the HR stratum was defined by (1) KMT2A-AFF1, (2) low hypodiploidy, (3) PPR, (4) FCM-MRD d15 HR, (5) IF, (6) PCR-MRD HR, and (7) patients with BCP-ALL and MRD load of ≥10^−3^ at EOI and any PCR-MRD positivity below 10^−3^ at EOC (TP2, slow early responders, SER). All patients with a negative MRD at EOI, irrespective of traditional VHR criteria, had no indication for HSCT. HSCT from an HLA matched donor (MD) only was indicated for patients with (1) PCR-MRD load of ≥10^−3^ and <10^−2^ at EOC or (2) low hypodiploid or KMT2A-AFF1 positive ALL and an MRD load of <10^−3^ not negative on TP2 [PCR-MRD, medium risk (MR)]. HLA matched or mismatched donor (MMD) HSCTs were indicated for patients with: IF or MRD load of ≥10^−2^ at TP2.

In the ongoing study AIEOP-BFM ALL 2017, the VHR subgroup with an indication for HSCT in non-infants is defined by (1) the presence of TCF3-HLF gene fusion, (2) KMT2A-AFF1 gene fusion, (3) hypodiploidy, (4) IKZF1^plus^ deletions with FCM-MRD d15 HR and SER, (5) PCR-MRD HR, and (6) T-ALL with PPR and/or FCM-MRD d15 HR and/or IF. Patients with MRD negativity at EOI are excluded from a HSCT indication. MMD-HSCTs are reserved for a PCR-MRD TP2 ≥5 × 10^−3^, all TCF3-HLF fused leukemias and those with IF.

### European ALLTogether1 Collaborative

The ALLTogether1 protocol (ATP) is the first clinical study (NCT03911128) designed by the collaborative ALLTogether consortium that consists of the study groups UKALL (UK), DCOG (the Netherlands), CoALL (Germany), BSPHO (Belgium), NOPHO (Sweden, Denmark, Norway, Finland, Iceland, Lithuania, and Estonia), SHOP (Portugal), PHOAI (Ireland), and SFCE (France) and represents 14 European countries (25). The scientific study questions, therapy elements, and risk-stratifications in ATP are based on the long-standing previous experience of the member study-groups in designing treatment protocols for ALL in childhood and young adults (AYA), and meticulously compiled and merged data from the groups' comparable previous study results. The ATP is open for enrollment of patients 1–45 years of age with newly diagnosed BCP-ALL and ALL of T-cell origin, including patients with Down syndrome (DS) and Ph-like genetic lesions but excluding patients with Ph+ ALL. The estimated total recruitment into the protocol is 6,430 patients over 5 years.

The ATP has defined the HR ALL group (estimated to constitute ~3% of all patients) based on specific criteria for age, cell of origin (BCP- or T-ALL), NCI-risk group and cytogenetics at diagnosis, and most importantly, the MRD response during therapy. MRD is measured by both multicolor FCM and PCR analysis of Ig/TCR gene rearrangements, and the highest MRD level determined by any of these methods will be used for therapy stratification. Time points for MRD analyses are at the EOI (TP1) and EOC (TP2); for the HR group, there is an additional mid-consolidation time point at day 50 (TP1.5).

In general, HR ALL patients <16 years of age are stratified to either high-risk chemotherapy (HR-chemo group) or additional consolidation by HSCT (HR-HSCT group). Patients ≥16 years at diagnosis with any HR criteria are stratified to the HR-HSCT arm.

More specifically, as of September 2021, the ATP has defined the following HR patient subgroups ≤18 years of age to be candidates for HSCT: (1) MRD ≥0.05% at EOC (TP2), (2) MRD ≥5% at EOI (TP1) and ≥0.5% at mid-consolidation day 50 (TP1.5); such patients are intensified with HR block therapy prior to HSCT unless aiming for the CAR-T cell option (CASSIOPEIA), and (3) t(17;19)(q22;p13)TCF3/HLF, irrespective of MRD levels at TP1, TP1.5, or TP2. Moreover, patients ≥16 years at diagnosis have a slightly broader HSCT indication in ATP, with additional criteria including: (4) MRD at TP1 ≥5% regardless subsequent MRD levels, (5) NCI high-risk disease at diagnosis and MRD at TP2 ≥0.01%, or (6) extramedullary disease not in CR1 at TP2. Of note, ATP patients who will enter CASSIOPEIA, but experience re-appearance of MRD and/or early B-cell recovery post-CAR-T cell infusion and do not respond to a re-infusion of tisagenlecleucel, will also have an HSCT indication. Finally, patients with BCR-ABL1-like genetic lesions receive a tyrosine kinase inhibitor (TKI) from day 15 of induction therapy and are eligible for HSCT if the MRD remains ≥0.05% at TP2.

### Hematopoietic Stem Cell Transplant for High-Risk ALLTogether1 Patients

Most transplantation centers in the ATP participate in the ALL SCTped 2012 FORUM trial (NCT01949129). The FORUM trial was set up to investigate the non-inferiority of a non-TBI-based conditioning for children ≥4 years of age with ALL and an HSCT indication compared with a standard TBI conditioning. The randomized part of the trial was prematurely terminated in 2019 due to a significantly higher EFS and OS in the TBI arm (26). For 224 patients transplanted in CR1, 2-year OS were 91 vs. 79% in the TBI arm and chemotherapy-conditioning arm, respectively. The trial is still open and enrolling patients, now non-randomly assigned to conditioning with TBI12Gy/VP16 (60 mg/kg) as the standard conditioning for children ≥4 years of age, to obtain data necessary to answer secondary endpoints of the study and study questions in the non-randomized cohorts. It is expected that the vast majority of HR ALL patients from ALLTogether1 who will be transplanted in CR1 will be enrolled into and follow the guidance of the FORUM protocol.

## Newly Diagnosed T-Cell Acute Lymphoblastic Leukemia

### Children's Oncology Group and Berlin–Frankfurt–Munster Groups

In the past, T-cell ALL (T-ALL) has generally been more difficult to treat, and outcomes were inferior to those with BCP-ALL. However, intensification of first-line therapy for T-ALL has improved the prognosis significantly with outcomes being nearly equivalent to that of BCP-ALL resulting in 5-year EFS and OS reaching 85 and 90%, respectively (1, 27–29). In T-ALL, the main factor for risk stratification during frontline therapy remains MRD assessment at the end of induction and at the end of consolidation (30). Moreover, there are different MRD kinetics between T-ALL and BCP-ALL in terms of time to achieve low or undetectable levels (31).

The COG defined very high-risk T-ALL as positive MRD at EOC ≥0.1% (NCT02112916) and evaluated whether such patients could attain MRD negative status after three cycles of intensive BFM blocks compared with alternative treatment such as HSCT; results are still anticipated. In comparison, the AIEOP-BFM group allocates patients with T-cell ALL to the high-risk group if they fulfill the following criteria: prednisone-poor response (PPR), FCM-MRD d15 HR, non-remission on day 33 and PCR-MRD on day 78 (TP2) ≥5 × 10^−4^. The prognostic value of MRD levels at the end of consolidation (day 78) was observed during the AIEOP-BFM-ALL 2000 study by analyzing the outcome of 464 patients with T-ALL. Although MRD negativity at day 33 was the most favorable prognostic factor, patients who turned negative only at day 78 (EOC) after being positive at day 33 had also an excellent outcome. The study concluded that MRD ≥10^−3^ at the EOC represents the most important predictive factor for relapse in childhood T-ALL (27).

The superiority of HSCT in CR1 for patients with HR T-cell ALL compared with chemotherapy alone was shown in a study analyzing the outcome of patients with T-cell ALL and high-risk features [defined by prednisone poor response (PPR) and non-remission on day 33], registered in the ALL-BFM 90 and ALL-BFM 95 trials in which the 36 children who received HSCT in CR1 had a 5-year DFS rate of 67% ± 8% vs. 42% ± 5% in the 120 patients treated with chemotherapy alone. The 5-year OS rate for the transplanted group was 67% ± 8% vs. 47% ± 5% in the chemotherapy arm (20). Balduzzi et al. reported the results of a prospective study on 357 children enrolled between 1995 and 2000 with newly diagnosed very high risk ALL including T-ALL with PPR or with PPR and high WBC ≥100,000/μl diagnosis, and randomized patients to HSCT based on an available HLA-matched related donor or chemotherapy. The results favored transplantation (5-year DFS was 56.7% in children assigned to transplantation as compared with 40.6% in those allocated for chemotherapy alone). Within the subset of patients with T-ALL, those allocated to transplant had a 5-year DFS of 62.4% compared with 54.3% in the chemotherapy arm. Moreover, children with T-ALL and PPR and high WBC counts receiving a transplant also had a better outcome than those with chemotherapy alone with DFS of 55.9 and 48%, respectively (19).

### Hematopoietic Stem Cell Transplant in Children With T-Cell-Acute Lymphoblastic Leukemia in First Complete Remission

**Figure 2:**
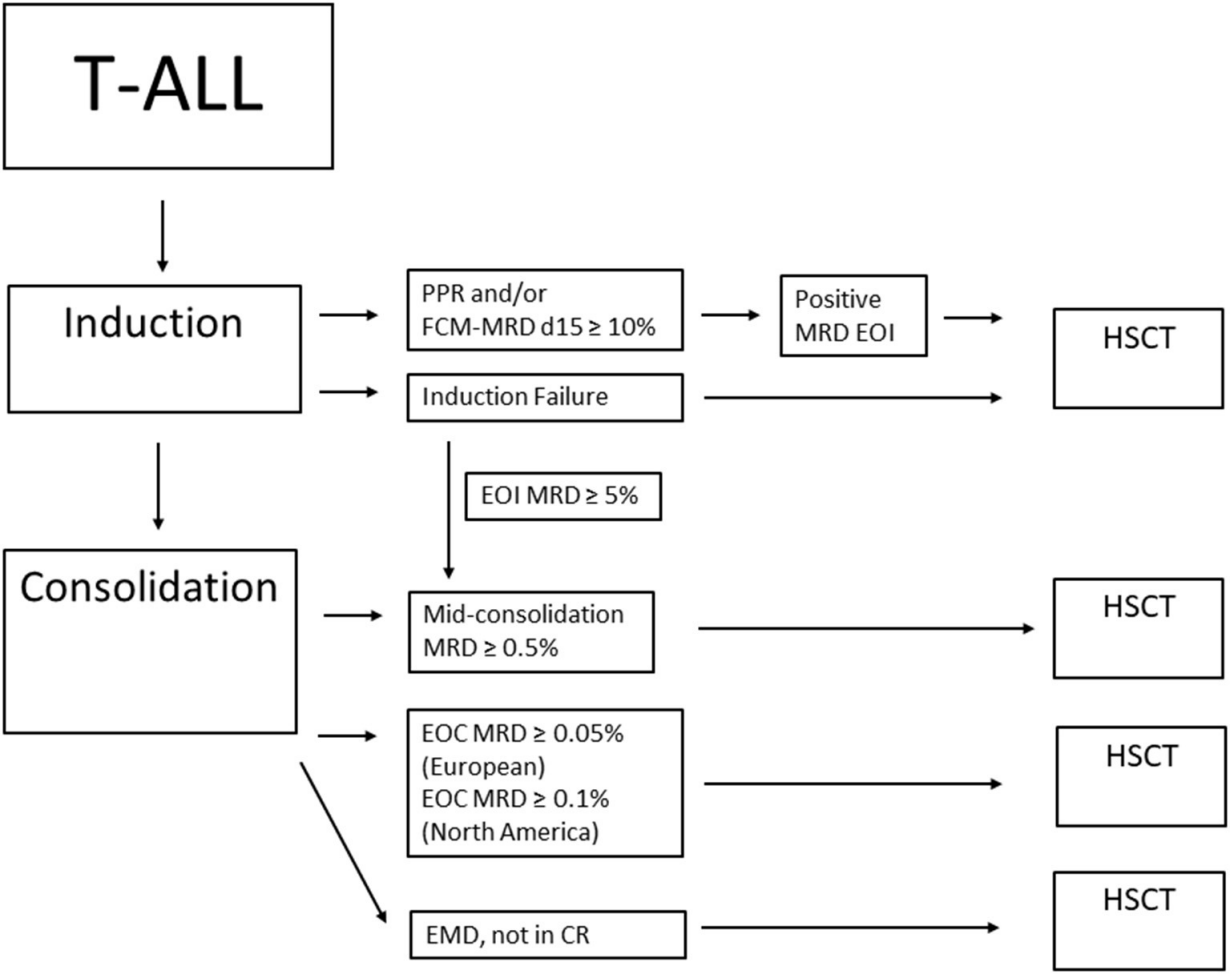
Summary of HSCT considerations for T-ALL in CR1. PPR, prednisone poor response; FCM-MRD d15, flow cytometry MRD on day 15; MRD, minimal residual disease; EOI, end of induction; EOC, end of consolidation; EMD, extramedullary disease; HSCT, hematopoietic stem cell transplant.

Children with T-ALL and high MRD levels at EOC are considered candidates to receive an HSCT in CR1 due to their probability of EFS of 50% or less and very poor outcome after an eventual relapse (32). The COG approach is based on MRD and patients with EOC MRD ≥0.1% are considered for HSCT in CR1 (30). In the ATP study, the current indication for HSCT in T-ALL include patients with the following: (1) MRD ≥5% at TP1 and MRD ≥0.5% at TP1.5 or (2) MRD ≥5% at TP1, MRD <0.5% at TP1.5, but detectable at TP2 or (3) MRD <5% at TP1, but MRD ≥0.05% at TP2, or (4) extramedullary disease, who are not in CR1 at TP2 (Figure 2).

### Hematopoietic Stem Cell Transplant for T-Cell-Acute Lymphoblastic Leukemia With Primary Induction Failure

Primary induction failure in T-ALL carries a very poor prognosis with 10-year OS of 28% (16). The study by Schrappe et al. showed a significant survival advantage after any HSCT in the T-ALL subset with a 10-year OS rate of 40% for matched related donor-HSCT and 45% for other types of allo-HSCT, compared with only 26% for patients allocated to chemotherapy alone, but this was prior to the incorporation of MRD in frontline trials (17). With MRD data from UKALL2003, the use of HSCT can be more defined in this population and has been recommended for patients with EOI MRD ≥5%, except for those under 16 years of age who achieved a EOC MRD <10^−4^ (17, 30).

## Relapsed Acute Lymphoblastic Leukemia: Risk Stratification and Reinduction Therapy

Survival following relapse of ALL remains poor. Risk stratification of relapsed ALL takes into account time from diagnosis to relapse, involvement of marrow ± extramedullary disease (EMD) and immunophenotype (5, 33, 34). Definitions of risk status at the time of relapse vary among collaborative groups (see Table 1), but overall, patients with a shorter time from diagnosis (North America) or end of treatment (Europe) to relapse, marrow involvement, and T-cell ALL have the worst prognosis (35–37).

**Table 1:** Risk stratification for acute lymphoblastic leukemia (ALL) in first relapse.

**Children's oncology group (35)**	**BFM group (36)**	**UK group (37)**	**IntReALL consortium**
Low Late B-ALL marrow, end-block 1 MRD <0.1% Late IEM, end-block 1 MRD <0.1%	Low (S1) Late IEM relapses	Standard Late IEM relapse	Standard (S1 and some S2) Early and late IEM relapses, of B-ALL or T-ALL Late B-ALL isolated marrow relapses Early/late B-ALL combined relapses
Intermediate Late B-ALL marrow, end-block 1 MRD ≥0.1% Late IEM, end-block 1 MRD ≥0.1%	Intermediate (S2) Early IEM relapses Late B-ALL isolated marrow relapses Early/late B-ALL combined relapses Very early IEM relapses	Intermediate Early IEM relapses Late B-ALL isolated marrow relapses Early/late B-ALL combined relapses	
High Early B-ALL marrow Early IEM T-ALL relapse, any site and timing	High (S3 and S4) Very early and early B-ALL marrow relapses Very early B-ALL combined relapses T-ALL marrow relapses (regardless of timing)	High Very early IEM relapse Very early and early B-ALL marrow relapses Very early B-ALL combined relapse T-ALL marrow or combined relapse, any timing	High (S3, S4 and some S2) All very early relapses, irrespective of site and phenotype Early B-ALL isolated marrow relapses T-ALL marrow relapses, combined or isolated (regardless of timing)

*COG definitions: IEM relapse (<18 months from diagnosis), late IEM (≥18 months from diagnosis); early marrow relapse (<36 months from diagnosis), and late marrow relapse (≥36 months from diagnosis)*.

*BFM and UK definitions: very early (<18 months from diagnosis), early (18 months from diagnosis but <6 months after end of treatment), and late (>6 months after end of treatment)*.

*IEM, isolated extramedullary disease; B-ALL, B-cell-acute lymphoblastic leukemia; MRD, minimal residual disease; BFM Group, Berlin–Frankfurt–Munster Group; T-ALL, T-cell-acute lymphoblastic leukemia*.

### Pre-hematopoietic Stem Cell Transplant Therapy for First Relapse of Pediatric Acute Lymphoblastic Leukemia

Prior to the availability of MRD monitoring by FCM or PCR, the goal of reinduction therapy for children with relapsed ALL was to attain a morphologic remission prior to proceeding to allogeneic HSCT as quickly as possible before the disease could recur. With continued improvements in the efficacy of frontline therapies, single courses of reinduction therapy for relapsed ALL became increasingly intensive, with a concomitant increase in treatment-related morbidity and mortality (TRM), sometimes precluding a patient from proceeding to HSCT. This led to strategies that incorporated multiple courses of reinduction therapy prior to HSCT to reduce TRM while further reducing pre-HSCT leukemic burden.

### Children's Oncology Group Re-induction Strategy for High-Risk Acute Lymphoblastic Leukemia Relapse

Seeking to improve the efficacy of re-induction therapy for first relapse of childhood ALL and to mitigate TRM associated with a single maximally intensive course of re-induction chemotherapy prior to HSCT, the Children's Oncology Group developed a strategy employing three sequential blocks of intensive chemotherapy to attain as “deep” a remission as possible prior to allogeneic HSCT, which could also serve as a reinduction “platform” to subsequently facilitate the evaluation of novel agents at first relapse of childhood ALL. COG AALL01P2, a pilot study evaluating the safety and efficacy of this approach with incorporation of MRD testing by flow cytometry at the end of each treatment block, demonstrated that second and third blocks of post-re-induction chemotherapy prior to HSCT resulted in further reduction of MRD levels in 40 of 56 patients who were MRD positive after block 1, with an acceptable (<5%) rate of TRM (38). Remission re-induction rates with this regimen were 68% for those with early relapse (<36 months from initial diagnosis) vs. 96% for those with late relapse. Patients with very early relapse (<18 months) fared poorly, with CR2 rates of only 45%. Of note, five of seven patients with T-cell ALL failed to attain remission. Post-induction therapy was at the discretion of the treating physician, precluding meaningful assessment of the impact of this re-induction regimen on long-term survival.

Subsequent studies built upon this re-induction platform concept with the introduction of novel agents including epratuzumab (a humanized monoclonal antibody targeting CD22) in ADVL04P2 and bortezomib (a proteasome inhibitor approved for use in multiple myeloma) in AALL07P1 into the re-induction platform established in AALL01P2 (38, 39). The overall CR2 rate in AALL07P1 with the addition of bortezomib for BCP-ALL patients <21 years of age with early relapse was 68%, not significantly different from the CR2 rate in AALL01P2 of 74%. In contrast, the CR2 rate in AALL07P1 for T-ALL patients in first relapse was 68% (15/22 patients)—a significant improvement over that seen in AALL01P2 with the same multi-agent chemotherapy regimen without bortezomib, as well as in a phase I/II trial of nelarabine, etoposide, and cyclophosphamide in first relapse of T-ALL, which achieved an overall response rate of 44% (40).

### Berlin–Frankfurt–Munster and UK Re-induction Strategies for High-Risk Acute Lymphoblastic Leukemia Relapse

The BFM ALL-REZ 2002 study employed a multi-course re-induction approach for first relapse of childhood ALL, consisting of a cytoreductive pre-phase with dexamethasone, followed by two poly-chemotherapy courses (F1/F2) over a period of 5 weeks and randomization of subsequent repetitive intensive chemotherapy blocks (R1 and R2 vs. IDA-II) for high-risk patients prior to HSCT at 12–18 weeks after the start of re-induction therapy (36). The ALL R3 study for first relapse of childhood ALL, conducted by the Children's Cancer and Leukemia Group in the UK and the Australian and New Zealand Children's Hematology/Oncology Group, randomized patients to receive either idarubicin or mitoxantrone as a component of a three-block re-induction regimen prior to HSCT for high-risk group patients and intermediate risk group patients with post-induction high MRD levels (37). Mitoxantrone conferred a significant benefit in progression-free and overall survival vs. idarubicin (64·6% vs. 35.9% PFS and 69·0% vs. 45.2% OS, respectively). A recent comparison of outcomes for patients treated on these two trials concluded “Improvements in outcomes for HR ALL relapses require novel compounds in induction therapy to improve remission rates” (41). Several novel immunotherapeutic agent have subsequently shown promise in studies of children with relapsed B-lineage ALL.

### The Emerging Role of Immunotherapies in Re-induction of Relapsed Acute Lymphoblastic Leukemia

Inotuzumab ozogamicin is a novel immunotoxin composed of a CD22-directed humanized monoclonal antibody conjugated to the intracellular toxin calicheamicin. In a randomized trial of adults with relapsed or refractory B-lineage ALL, inotuzumab ozogamicin was associated with higher CR and MRD-negative rates, as well as longer progression-free and overall survival, than standard chemotherapy; however, veno-occlusive liver disease (VOD) was a major adverse event associated with inotuzumab ozogamicin, raising concerns about increased risk for VOD with subsequent HSCT (42). The published retrospective experience in relapsed childhood ALL with inotuzumab ozogamicin is limited, but suggests a similar safety and efficacy profile as in adults (43, 44); prospective trials of inotuzumab ozogamicin for re-induction of relapsed/refractory pediatric BCP-ALL are ongoing (NCT02981628, EudraCT 2016-000227-71).

The COG evaluated the role of another novel agent, the anti-CD19 bispecific T cell–engaging antibody construct blinatumomab, by randomizing patients with first relapse of B-lineage ALL and high-risk features (any early relapse <36 months, or later relapse with MRD >0.1% by FCM following 4 weeks of intensive chemotherapy based on UK ALL R3 mitoxantrone Block 1 therapy) to receive two subsequent blocks of intensive chemotherapy modeled upon the remaining courses of UK ALL R3 re-induction chemotherapy or two courses of blinatumomab, prior to protocol-defined HSCT (35). Although the stopping rule for disease-free survival efficacy in this trial was not met, the study's data and safety monitoring committee recommended early closure of randomization due to a combination of higher disease-free survival and overall survival, lower rates of serious toxicity, and higher rates of MRD clearance for blinatumomab relative to chemotherapy. The 2-year disease-free survival and overall survival rates for high-risk patients receiving blinatumomab on the study were 54.4 and 71.3%, respectively. Importantly, 70% in the blinatumomab group proceeded to HSCT, compared with 43% for the chemotherapy group, suggesting that blinatumomab's enhanced safety profile pre-HSCT as compared with that of intensive chemotherapy was an important element of its success. A similar European randomized trial of post-re-induction blinatumomab in children with high-risk first relapse of B-lineage ALL, in which 1 block of consolidative chemotherapy was replaced with a course of blinatumomab prior to HSCT, demonstrated improvements in EFS, MRD reduction, and incidence of serious adverse events with blinatumomab compared with conventional chemotherapy (45). Given the significant TRM associated with multi-agent re-induction chemotherapy in older patients in this COG trial (46), the current COG trial for first relapse of childhood ALL is exploring the efficacy of blinatumomab alone vs. blinatumomab and nivolumab as re-induction therapy, with a reduced intensity chemotherapy “prephase” reserved for selected subsets of patients with higher risk features (NCT04546399).

CAR T-cells are another type of T-cell redirecting therapy with significant activity against relapsed and refractory childhood ALL. In a phase 1 study of a CAR T-cell targeting CD19 and containing a CD28 costimulatory domain, Lee et al. showed that children and young adults with heavily pretreated B-lineage ALL receiving this agent achieved a 70% CR, with 12/20 (60%) of patients attaining an MRD-negative CR (47). All 10 patients with an MRD-negative CR who subsequently underwent HSCT remained in remission at the time of the report, while 2 patients without subsequent HSCT both relapsed within 6 months. A modified version of this agent is currently in an international multicenter phase I/II trial for relapsed pediatric ALL as bridging therapy prior to HSCT (NCT02625480).

A CAR T-cell targeting CD19 and containing a 41BB costimulatory domain developed at Seattle Children's Research Institute produced a 93% MRD-negative remission rate in children and young adults receiving an infusion and a 100% MRD-negative remission rate in the subset of patients who received fludarabine and cyclophosphamide lymphodepletion prior to infusion (48). The estimated 12-month EFS and OS of infused patients were 50.8 and 69.5%, respectively. Eleven patients underwent consolidative allogeneic HSCT, with two subsequently experiencing recurrence at the time of publication.

In a global phase II study sponsored by Novartis, a CAR T-cell targeting CD19 with a 41BB costimulatory domain developed at the University of Pennsylvania produced an MRD-negative CR rate of 81% in children and young adults receiving an infusion (10). The rates of event-free survival and overall survival were 50 and 76%, respectively, at 12 months. This agent, tisagenlecleucel, was subsequently approved by the Food and Drug Administration (FDA), Health Canada and the European Medicines Agency (EMA) for pediatric and young adult patients up to 25 years of age with B-lineage ALL that is refractory, in relapse after transplant, or in second or later relapse.

It is noteworthy that all studies (except for ZUMA-4) were conducted in anti-CD19 therapy-naïve patients, and thus, the outcome in patients receiving CAR-T after anti-CD19 immunotherapy is unknown. Since blinatumomab is more accessible and affordable than CAR-T, many children receive blinatumomab prior to CAR-T.

All of the aforementioned T-cell redirecting therapies are associated with varying incidence and degrees of cytokine release syndrome (CRS) and immune effector cell-associated neurotoxicity syndrome (ICANS). The high rates of complete response and MRD negativity associated with CD19-directed CAR T cells make them a compelling first choice for re-induction of multiple relapsed ALL in children and young adults. The role of HSCT following successful re-induction therapy with CAR T-cells depends on a number of factors, including the duration of CAR T-cell persistence, which is influenced by the specific costimulatory element(s) present in each construct, and other factors which have not been fully identified to date. Thus, the role of HSCT following induction of remission with CAR T-cell therapy in multiple relapsed childhood B-lineage ALL has not yet been clearly established and is further explored in detail in another manuscript in this issue.

### Hematopoietic Stem Cell Transplant in Second Remission for Relapsed B-Acute Lymphoblastic Leukemia

The introduction of novel immunotherapy strategies has improved re-induction rates in relapsed ALL while minimizing toxicity, raising questions about which patients can be treated with these approaches alone and which need consolidation with HSCT. Within COG trials, high-risk relapse include those with very early isolated extramedullary relapse (<18 months from diagnosis) and early marrow relapse (<36 months from diagnosis); however, the incorporation of MRD after re-induction in AALL0433 has been shown to be highly prognostic and is used to guide HSCT decisions (49). Relapsed B-ALL patients who achieved MRD <0.1% after Induction-1 had a superior EFS and OS of 85 and 94% vs. 54 and 61%, respectively, for patients with MRD ≥0.1%. In this study, less than one-third of all patients were treated with HSCT (*n* = 74) and had an improved disease-free survival (DFS) of 78 vs. 67% compared with chemotherapy, but similar OS of 82–86%. When analyzed by MRD, those with MRD <0.1% at the end of Induction-1 had a trend toward improved DFS with HSCT over chemotherapy but again with similar OS, whereas those with MRD >0.1% had no benefit from HSCT in DFS or OS. Currently, an MRD level of <0.01% after re-induction with chemotherapy or blinatumomab is recommended for consideration of HSCT (50). Transplant indications among the various cooperative study groups are summarized in Table 2.

**Table 2:** Current indications for hematopoietic stem cell transplant (HSCT) by the cooperative study group.

***B-ALL Newly Diagnosed***	**COG**	**BFM-AIEOP**	**ALLTogether1**
Hypodiploid ALL	Positive EOC-MRD	Positive EOC-MRD	As below, according to MRD
Induction Failure	Positive EOC-MRD	Positive EOC-MRD	
Positive MRD	NCI HR: EOC MRD any value NCI SR: EOC MRD ≥1%	All PCR-MRD ≥5 × 10^−4^ at EOC	*All patients ≤ 18 years of age:* MRD ≥0.05% at EOC (TP2) or MRD ≥5% at EOI (TP1) and ≥0.5% mid-consolidation day 50 (TP1.5) unless targeting for CASSIOPEIA MRD re-appearance (early B-cell recovery following CAR T cell (re-)infusion in CASSIOPEIA *Additionally, in patients ≥16 years at diagnosis:* MRD at TP1 ≥5% regardless of subsequent MRD levels or NCI high-risk disease at diagnosis and MRD at TP2 ≥0.01% or Extramedullary disease not in CR1 at TP2
*t(17;19)* TCF3-HLF		All cases of TCF3-HLF, irrespective of MRD	All cases, irrespective of MRD levels at TP1, TP1.5 or TP2
*IKZF1^*plus*^*		*IKZF1^*plus*^* and FCM MRD d15 ≥10% and PCR-MRD EOI pos, EOC pos <5 × 10^−4^ *IKZF1^*plus*^* and FCM MRD d15 <10% and EOC ≥5 × 10^−4^	As above, according to MRD
***T-ALL Newly Diagnosed***	**COG**	**BFM- AIEOP**	**ALLTogether1**
	Positive EOC MRD ≥0.1% T-ALL with PIF	T-ALL: PPR and/or FCM-MRD d15 ≥10% with either: PCR-MRD positive at EOI, or EOC MRD ≥5 × 10^−4^	MRD ≥5% at TP1 and MRD ≥0.5% at TP1.5 or MRD ≥5% at TP1, MRD <0.5% at TP1.5, but detectable at TP2 or MRD <5% at TP1, but MRD ≥0.05% at TP2 or Extramedullary disease, who are not in CR1 at TP2
***ALL Relapse***	**COG**	**IntReALL**	
	Marrow relapse: early or late with MRD >0.1% IEM relapse: early or late relapse with MRD >0.1% T-cell ALL: any relapse	All HR relapse (see IntReALL risk groups in Table 1) SR relapse with positive MRD EOI, or early isolated EM relapse if MD available	
***Special Groups***			
***Infant ALL***	**COG**	**Interfant group**	
	KMT2A-AFF1 rearrangement and positive EOC-MRD	*Interfant-06 criteria: KMT2A*-rearranged and age <6 months at diagnosis with either WBC ≥ 300,000/μl or PPR	
***Ph+** **ALL***	**COG**	**EsPhALL**	
	Positive EOC-MRD	*Current EsPhALL criteria:* EOC MRD ≥5 × 10^−4^ (high positive) or <5 × 10^−4^ (low positive) at EOC and still positive at any level at end of HR block 3	
***MPAL***	**COG**	**BFM- AIEOP**	**I-BFM AMBI 2018**
	Positive EOC-MRD	Positive EOC-MRD	No CR at defined time points during ALL or AML therapy

*IEM, isolated extramedullary disease; MPAL, mixed phenotype acute leukemia; PIF, primary induction failure; COG, Children's Oncology Group; EOC, end of consolidation; NCI, National Cancer Institute; HR, high risk; SR, standard risk; BFM-AIEOP, Berlin–Frankfurt–Munster–Associazione Italiana Ematologia Oncologia Pediatrica protocol; PCR, polymerase chain reaction; FCM, flow cytometry; MD, matched donor; EsPhALL, European study for pediatrics Ph+ ALL; CR, complete remission; CR1, first complete remission; TP1, time point 1; TP2, time point 2; CAR, chimeric antigen receptor*.

In the last 30 years several prospective trials have been performed in Europe in children with relapsed ALL by different cooperative groups (e.g., AIEOP, ALL-REZ, BFM, COPRALL, UKALLR) (33, 51–53). Since 1995, the ALL-REZ BFM group used this strategy to risk stratify patients into four treatment groups, termed S1–S4 (36, 54). In the AIEOP LALREC2003, patients in the S3 and S4 risk groups had an indication to HSCT irrespective of response to induction therapy and donor availability; S2 patients had an indication to HSCT if they had an HLA-identical family donor available; in case this donor was not available, response to therapy at time point 3 (i.e., after 12 weeks of treatment) defined the indication for matched unrelated donor HSCT.

Thus, under the umbrella of the International BFM Study Group (I-BFM SG), the Resistant Disease Committee designed an international study for treatment of childhood relapsed ALL, IntReALL 2010, with two risk-groups instead of four: standard risk (defined as all early and late B-ALL relapses (except for early BM isolated relapses) and early and late T-ALL isolated extramedullary relapses) and high-risk relapses (i.e., all very early relapses, irrespective of phenotype and site of relapse, all T-ALL relapse with bone marrow involvement, early B-ALL BM isolated relapses). The treatment strategy with curative intent in this population was to induce a second CR with conventional intensive chemotherapy and then consolidate this with HSCT in all HR patients and some SR patients. For SR patients, indication to HSCT was defined by MRD evaluation at EOI. However, since patients were randomized to receive either BFM-like or UKALL-like induction, the MRD cut-off (as well as time of evaluation) depends on the induction intensity of the respective treatment arm: patients of REZ BFM 2002 arm are eligible for HSCT if MRD at EOI is ≥10^−3^, while patients treated in the UK-ALLR3 arm are eligible for HSCT if MRD after induction is ≥10^−4^. Moreover, patients with an isolated early EM relapse were also stratified to HSCT if a matched donor was available.

### Hematopoietic Stem Cell Transplant in Second Remission for Relapsed T-Cell-Acute Lymphoblastic Leukemia

The majority of patients with T-ALL relapse will do so within 2 years after initial diagnosis and unfortunately prognosis is poor, with a survival rate of only 25% (34, 55). Immunophenotype has major prognostic significance with several studies demonstrating that relapsed T-ALL carries a much worse prognosis compared with B-ALL (5, 56, 57). Due to the poor outcome of patients with T-cell ALL relapse treated by chemotherapy alone, HSCT has become the standard approach in most collaborative groups. However, the success of HSCT depends on the remission-re-induction rates to salvage therapy. Although, historically, only about 30% of patients achieved CR2, the addition of nelarabine and more recently of the proteasome inhibitor bortezomib was associated with remission rates of 44% and even 68%, respectively (39, 40).

Barrett et al. compared the results of MSD transplantation in 376 children registered in the IBMTR with those of 540 children treated by the Pediatric Oncology Group and showed that children with T-ALL in CR2 had a 5-year leukemia-free survival of 32% in the transplantation arm vs. 11% in the chemotherapy arm (57). Based on the rather low number of patients at the time of analysis, clear conclusions with regard to this subset of patients could not be drawn. A population-based report of the Austrian BFM Study Group compared the survival rates after HSCT with those after chemotherapy in 203 ALL patients with recurrent disease registered in consecutive BFM trials in Austria between 1981 and 1999. The outcome analysis of the subset of patients with T-phenotype showed that four of the six survivors received HSCT suggesting a benefit of transplantation over chemotherapy alone, but the numbers were small (58). In a long-term outcome analysis of the ALL-REZ BFM 90 study, Tallen et al. showed that EFS in the HR group, which included children with any relapse of T-ALL, was significantly higher in patients allocated to transplantation than in those with chemo-radio therapy alone (54). Multivariate analysis showed that immunophenotype and HSCT (as a time-dependent covariate) in the HR group were independent predictors of EFS. In contrast, patients with late extramedullary relapse of T-ALL had significantly better results being, therefore, no longer allocated to the HR group in the subsequent BFM trials. A retrospective analysis of CIBMTR data performed by Burke et al. on 229 patients with relapsed T-cell ALL who received HSCT between 2000 and 2011 revealed a 3-year OS and 3-year DFS rates of 48 and 46%, respectively (59). Multivariate analysis confirmed that patients with bone marrow with or without extramedullary relapse portend a much higher risk of relapse compared with isolated extramedullary relapse, confirming the results reported by Tallen et al. The authors conclude that the use of HSCT in pediatric patients with relapsed T-cell ALL in CR2 is warranted. According to the IntReALL2010 protocol, all patients with a very early isolated extramedullary relapse of T-cell ALL or with any bone marrow relapse of T-cell ALL have an indication for HSCT in CR2, both criteria being considered HR features (32).

## Special Groups

### Infant Acute Lymphoblastic Leukemia

Historically, infants with *KMT2A*-rearranged leukemia had very poor outcomes with 5-year event-free survival ranging from 13 to 33% and HSCT in CR1 was often used to consolidate remission (60, 61). However, studies from both COG (CCG 1953 and POG 9407) and Europe (Interfant-99) indicated that with intensified chemotherapy, outcomes were similar to those treated with HSCT, both in the range of 50% EFS (62, 63). Although no clear indication for HSCT exist within the COG for infant ALL, the highest risk group (*KMT2A*-rearranged and <3 months of age at diagnosis) have the worst survival of 20%, and HSCT is often recommended in CR1, for those with positive MRD (64). In Interfant-99, a subset of infants with *KMT2A*-rearranged B-ALL who had two additional poor prognostic factors, age <6 months and either PPR at day 8 or an initial WBC ≥300,000/μl, benefited from HSCT over chemotherapy alone (5-year OS 66 vs. 20%) (65). In the Interfant-06 study, high-risk patients (defined as *KMT2A*-rearranged and age <6 months at diagnosis with either WBC ≥300,000/μl or PPR) were eligible for HSCT if they had an HLA-identical MSD or matched unrelated donor (66). Patients with medium-risk (MR group, defined as all others except for *KMT2A* germline) received HSCT if MRD was ≥10^−4^ at the start of OCTADA(D), due to poor outcomes in Interfant-99 (67). Although Interfant-06 was not designed to compare HSCT vs. chemotherapy, the HR group that eventually received HSCT, representing a selected population who did not suffer early relapse, had a 4-year DFS of 44%, while the MR group had a dismal outcome of 19% (66). Thus, HSCT remains restricted to the HR group and those that relapse after frontline therapy.

### Down Syndrome Patients

Patients with Down syndrome (DS) have a poor prognosis with considerable risk of TRM on intensified relapse chemotherapy protocols (68–70). In contrast, relapse was the main cause of treatment failure after HSCT in the pre-immunotherapy era (71). Among the various cooperative groups, DS patients with B-ALL stratified as high-risk are receiving immunotherapy approaches in an attempt to improve disease response while minimizing TRM. In an upfront COG study for newly diagnosed B-ALL patients (NCT03914625), DS patients who meet the NCI-HR criteria or have MRD ≥0.01% at EOI (or ≥0.1% for double trisomies of 4 and 10) are assigned to receive three cycles of blinatumomab in combination with a less intensive chemotherapy backbone. Those who have positive EOC MRD ≥0.01% or have consolidation failure (≥1%) may have traditionally been treated with HSCT, but are currently eligible for CAR-T cell treatment on the CASSIOPEIA trial.

In the ATP, DS patients with MRD of ≥5% at EOI (TP1) are classified as HR DS patients and alternative immunotherapy-based approaches and/or modified HR treatment elements are used with the aim to achieve deeper and continuous remissions but to avoid block therapy and HSCT. Consolidation 1 is prolonged to last over 11 weeks (“augmented” BFM consolidation). HR DS patients with BCP-ALL are also eligible to participate in the ALLTogether1 sub-protocol for DS patients (“Phase II study to evaluate the efficacy and safety of blinatumomab in children and young adults with Down Syndrome and intermediate or high-risk BCP-ALL,” NCT04307576) and receive two cycles of blinatumomab substituting for the first half of the prolonged consolidation. Patients who do not adequately respond to blinatumomab or, alternatively, to the prolonged (11 weeks) HR consolidation (i.e., MRD ≥0.01% at TP2) will either (1) receive more blinatumomab and chemotherapy, or (2) be offered CAR T cell therapy (in CASSIOPEIA, if MRD is <5% at EOC and patient fulfills other eligibility criteria; see below), or (3) if MRD ≥1% at EOC or ≥5% mid-consolidation be counted as protocol therapy failure and offered suitable experimental therapy. In summary, the ATP does not stratify HR DS patients to HSCT during front-line therapy. Due to the substantial TRM experienced by patients with DS during conventional relapse chemotherapy, there is a growing number of patients with DS who are treated with CAR T-cell therapy already for a first relapse of CD19-positive BCP-ALL (72, 73).

### Mixed Phenotype Acute Leukemia

Patients with mixed phenotype acute leukemia (MPAL) are typically treated as per high-risk ALL. A central review of 54 MPAL cases within the COG showed a 5-year event free survival (EFS) and overall survival (OS) of 72 and 77%, respectively, among patients treated with ALL-directed chemotherapy, as opposed to acute myeloid leukemia chemotherapy, without the need for HSCT (74). When HSCT was compared with chemotherapy, there was a higher but statistically non-significant improvement in EFS (80 vs. 68%, *p* = 0.225); however, the 5-year OS was similar in both groups (80 vs. 75%). An international study led by the BFM group showed that survival in patients with ambiguous lineage leukemia was higher with ALL-type therapy than with AML-type therapy and that HSCT did not provide an overall benefit in this patient population (75). Therefore, current data indicate that MPAL in CR1 is best treated with ALL-based chemotherapy, except when the blasts harbor AML-specific gene fusions, are CD19-negative, and have no other lymphoid markers; in such patients, AML therapy is proposed (75). A current COG trial (AALL1732, NCT03959085) is testing the value of HSCT in those with MPAL and IF, EOI MRD >5% or EOC MRD >0.01%.

### CASSIOPEIA: Substituting Hematopoietic Stem Cell Transplant by Chimeric Antigen Receptor T-Cell Therapy in High-Risk B-Cell Precursor-Acute Lymphoblastic Leukemia

The CASSIOPEIA CAR T-cell protocol (NCT03876769) is an international multicenter phase II trial for *de novo* NCI-high risk BCP-ALL patients aged 1–25 years who are MRD+ by FCM (MRD ≥0.01%) at the EOC available in North America and Europe. The protocol is designed as a single-arm study evaluating safety and efficacy of tisagenlecleucel in HR BCP-ALL EOC MRD+ patients with 5-year disease-free survival as primary endpoint and a historic COG HR BCP-ALL cohort [protocol AALL0232 (15)] as comparator for outcome. For this reason, disease eligibility criteria for AALL0232 have been mirrored in CASSIOPEIA and, therefore, include only NCI high-risk patients (while excluding NCI low-risk) and only patients having received ALL therapy with a four-drug induction (except DS patients in need of modified induction) and a BFM-like consolidation/phase 1b. Patients with hypodiploid leukemia, t(9;22) and/or prior tyrosine kinase inhibitor therapy (as given to BCR-ABL1-like patients) are excluded. Patients with M3 BM at EOI or M2/M3 BM at EOC are neither eligible, as they have refractory ALL disease, which is not the scope of CASSIOPEIA but has recently been addressed in the ELIANA protocol (NCT02435849) (10).

In summary, a fraction of HR BCP-ALL patients, including patients with DS, have the option to enter the CASSIOPEIA trial if they fulfill study eligibility criteria including those mentioned previously. Those patients undergo leukapheresis when MRD positivity is confirmed by centralized MRD assessment, and receive interim maintenance with HD-MTX while awaiting CAR T cell manufacturing. Patients who remain MRD negative following a single infusion of tisagenlecleucel do not undergo consolidative HSCT (CAR T as stand-alone therapy); patients with early B-cell recovery and/or MRD appearance have the option of tisagenlecleucel re-infusion (76). Only patients who fail tisagenlecleucel therapy (± re-infusion) proceed to HSCT.

## Conclusion

Indications for HSCT have drastically evolved over the last two decades based on several advancements in the treatment of pediatric ALL: (1) intensification of therapy for those subtypes of ALL with a high risk of relapse, (2) inclusion of novel agents in upfront treatment (e.g., TKI's for Ph+ ALL and nelarabine for T-ALL), (3) incorporation and refinement of MRD to assess disease response, and (4) recent introduction of novel immunotherapies and immune effector cells. Despite the overall improvement in survival of *de novo* ALL and relapsed ALL, HSCT remains a necessary tool for consolidation in patients with the most resistant forms of the disease. Response to frontline therapy remains the best predictor of outcome, and the use of HSCT in CR1 is guided by MRD evaluation. Those patients with standard risk disease and poor response to treatment are treated with immunotherapeutic approaches, while those with high-risk disease are generally consolidated with HSCT. In the relapsed setting, MRD has also shown to be highly valuable and can identify patients who require HSCT in CR2 or can be treated with chemotherapy alone. Novel methods of disease response assessment include detection of MRD using next-generation sequencing (NGS) to detect MRD more deeply to the 10^−7^ level (77). These techniques are currently being evaluated prospectively in an upfront standard-risk COG trial (NCT03914625), the Pediatric Bone Marrow Transplant Consortium study (EndRAD, NCT03509961), and the EuroClonality-NGS consortium (78).

Both intensification of upfront therapy and the incorporation of novel immunotherapy in frontline studies have challenged the indications for transplant, which is now retained for those who have the most resistant diseases. Results from these current trials are highly anticipated and will inform whether the delayed application of HSCT will continue to improve patient outcomes while minimizing toxicity. In the era of immunotherapy, future challenges and goals will be to identify those who will require transplant for long-term cure, ascertain the appropriate timing of transplant in relation to novel immunotherapeutic approaches, and harmonize HSCT practices so that we can all learn from our collective experience.

## Author Contributions

All authors listed have made a substantial, direct, and intellectual contribution to the work and approved it for publication.

## Conflict of Interest

The authors declare that the research was conducted in the absence of any commercial or financial relationships that could be construed as a potential conflict of interest. The handling Editor declared a past co-authorship with several of the authors JB and PM.

## Publisher's Note

All claims expressed in this article are solely those of the authors and do not necessarily represent those of their affiliated organizations, or those of the publisher, the editors and the reviewers. Any product that may be evaluated in this article, or claim that may be made by its manufacturer, is not guaranteed or endorsed by the publisher.
